# Neurodevelopmental outcome of Italian preterm ELBW infants: an eleven years single center cohort

**DOI:** 10.1186/s13052-022-01303-9

**Published:** 2022-07-19

**Authors:** Camilla Caporali, Stefania Longo, Giovanna Tritto, Gianfranco Perotti, Camilla Pisoni, Cecilia Naboni, Barbara Gardella, Arsenio Spinillo, Federica Manzoni, Stefano Ghirardello, Renato Borgatti, Simona Orcesi, ELBWI Neurodevelopmental Follow-up Study Group, Ivana Olivieri, Roberta La Piana, Davide Tonduti, Alice Decio, Claudia Ravelli, Sara Olivotto, Giada Ariaudo, Silvia Spairani, Tiziana Figar

**Affiliations:** 1 Department of Brain and Behavioural Sciences, University of Pavia, Pavia, Italy; 2 Child Neurology and Psychiatry Unit, IRCCS Mondino Foundation, Pavia, Italy; 3 Neonatal Intensive Care Unit, Fondazione IRCCS Policlinico San Matteo, Pavia, Italy; 4 Fondazione Stella Maris Mediterraneo, Chiaromonte, Potenza Italy; 5 Department of Obstetrics and Gynecology, IRCCS Foundation Policlinico San Matteo and University of Pavia, Pavia, Italy; 6 Department of Clinical, Surgical, Diagnostic and Paediatric Sciences, University of Pavia, Pavia, Italy; 7 Biometry and Clinical Epidemiology, Fondazione IRCCS Policlinico San Matteo, Pavia, Italy; 8 Health Promotion - Environmental Epidemiology Unit, Hygene and Health Prevention Department, Health Protection Agency, Pavia, Italy; 9 Present Address: IRCCS Fondazione Don Carlo Gnocchi Onlus, Milan, Italy; 10 Present Address: Department of Neurology and Neurosurgery, Montreal Neurological Institute, McGill University, Montreal, QC Canada

**Keywords:** Prematurity, ELBWI, Neurodevelopment, Perinatal complications, NICU

## Abstract

**Background:**

Preterm extremely low birth weight infants (ELBWi) are known to be at greater risk of developing neuropsychiatric diseases. Identifying early predictors of outcome is essential to refer patients for early intervention. Few studies have investigated neurodevelopmental outcomes in Italian ELBWi. This study aims to describe neurodevelopmental outcome at 24 months of corrected age in an eleven-year single-center cohort of Italian ELBWi and to identify early risk factors for adverse neurodevelopmental outcomes.

**Methods:**

All infants born with birth weight < 1000 g and admitted to the Neonatal Intensive Care Unit of the “Fondazione IRCCS Policlinico San Matteo” hospital in Pavia, Italy, from Jan 1, 2005 to Dec 31, 2015 were eligible for inclusion. At 24 months, Griffiths’ Mental Developmental Scales Extended Revised (GMDS-ER) were administered. Neurodevelopmental outcome was classified as: normal, minor sequelae (minor neurological signs, General Quotient between 76 and 87), major sequelae (cerebral palsy; General Quotient ≤ 75; severe sensory impairment). Univariate and multivariate multinomial logistic regression models were performed to analyze the correlation between neonatal variables and neurodevelopmental outcome.

**Results:**

176 ELBWi were enrolled (mean gestational age 26.52 weeks sd2.23; mean birthweight 777.45 g sd142.89). 67% showed a normal outcome at 24 months, 17% minor sequelae and 16% major sequelae (4.6% cerebral palsy on overall sample). The most frequent major sequela was cognitive impairment (8.52%).

In the entire sample the median score on the Hearing-Speech subscale was lower than the median scores recorded on the other subscales and showed a significantly weaker correlation to each of the other subscales of the GMDS-ER.

Severely abnormal cUS findings (RRR 10.22 p 0.043) and bronchopulmonary dysplasia (RRR 4.36 p 0.008) were independent risk factors for major sequelae and bronchopulmonary dysplasia for minor sequelae (RRR 3.00 p 0.018) on multivariate multinomial logistic regression.

**Conclusions:**

This study showed an improvement in ELBWI survival rate without neurodevelopmental impairment at 24 months compared to previously reported international cohorts. Cognitive impairment was the most frequent major sequela. Median scores on GMDS-ER showed a peculiar developmental profile characterized by a selective deficit in the language domain. Severely abnormal cUS findings and bronchopulmonary dysplasia were confirmed as independent risk factors for major sequelae.

**Supplementary Information:**

The online version contains supplementary material available at 10.1186/s13052-022-01303-9.

## Background

In the last decades, the global increase in preterm birth rate and the concurrent reduction in mortality rate has led to a growing number of children who survived neonatal complications of preterm birth [1, 2]. Nevertheless, preterm infants, particularly those with birthweight below 1000 g (Extremely Low Birth Weight, ELBW), remain at greater risk of developing a broad spectrum of neuropsychiatric diseases through childhood and beyond [3, 4]. Many studies worldwide have investigated short-term neurodevelopmental outcomes of ELBW infants born in the 1990s and early 2000s [5–8]. However, few reported results and contribution to neuro-disability burden of extremely preterm infants born near the limit of viability in the last fifteen years [9–11].

Reports of the neurodevelopmental outcome of large prospective Italian ELBW infants’ cohorts still lack [12]. Therefore, these reports and the contribution of pre/perinatal and environmental factors are essential for early identification of subjects at greater risk of neurodevelopmental impairment and promptly starting early intervention programs targeted on infants and their families [13]. Therefore, this study aims to describe neurodevelopmental outcomes at 24 months of corrected age (CA) of an Italian cohort of ELBW preterm infants from a single Italian NICU and correlations with obstetric and neonatal factors.

## Methods

### Study population

**Fig. 1:**
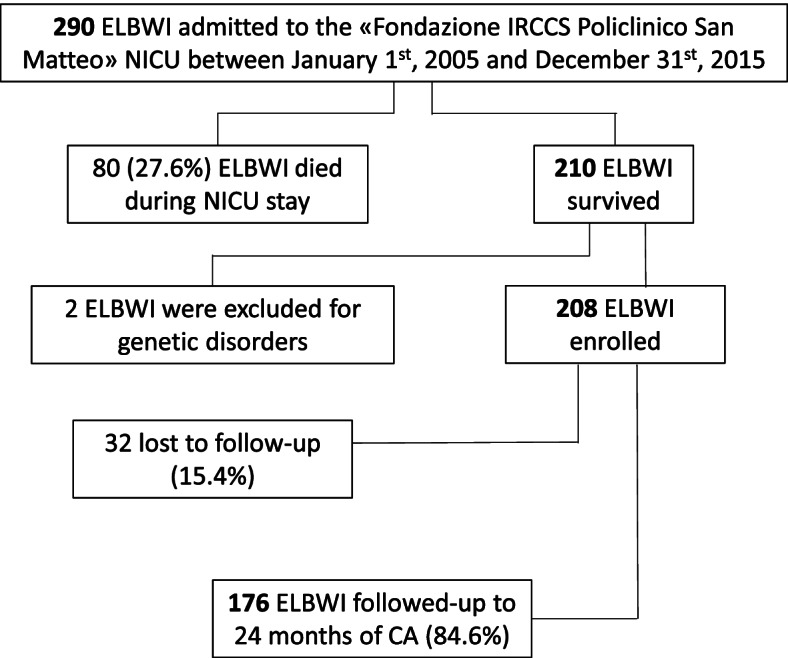
Enrollment flow-chart

All consecutive infants born with birth weight < 1000 g and admitted within 6 h from birth to the neonatal intensive care unit (NICU) of the “Fondazione IRCCS Policlinico San Matteo” hospital in Pavia, Italy, from Jan 1, 2005, to Dec 31, 2015 were eligible for inclusion (Fig. 1). All patients with congenital malformations and/or genetic disorders were excluded. The study was approved by the Ethical Committee Area Vasta Pavia—Fondazione IRCCS Policlinico San Matteo.

### Data collection

We recorded obstetric, perinatal and neonatal data for every newborn enrolled according to the Vermont Oxford Network (VON) database collection criteria [14]. The clinical risk index for babies (CRIB) score was calculated as previously described [15]. The time needed to recover from postnatal weight loss was recorded as the number of days required to restore birth weight.

### Ultrasound scan

Per institutional protocol, serial cerebral ultrasound (cUS), was performed in all preterm infants. All infants born before 35 weeks GA underwent cerebral cUS evaluation within the first 24 h of life. In infants born before 30 weeks GA, cUS was performed at 24, 48 and 72 h of life, at 7 days of life, then weekly up to 31 weeks post-menstrual age (PMA) and then at 36 and 40 weeks PMA. In infants born between 30 and 33 weeks GA, a first evaluation was performed within the first week of life, a second one at 3 weeks, and then at 40 weeks PMA [16]. Intraventricular hemorrhage (IVH), periventricular leukomalacia (PVL) and ventricular dilatation (VD) were classified according to Papile et al., de Vries et al., and to Ment et al. [17–19], respectively.

Following the Rademaker classification, cUS scans performed at 40 weeks of postmenstrual age (PMA) were classified as “normal”, “slightly abnormal” in the presence of grade I/II IVH, grade I PVL, germinal layer necrosis, or a combination of these features, or isolated VD; “severely abnormal” cUS scans was defined in the presence of grade III/IV IVH, cystic grade II/III PVL, thalamic lesions, focal infarction, or post-hemorrhagic VD needing surgical intervention [20].

### Neurological and neurodevelopmental assessment and follow up

A child neuropsychiatrist, unaware of the cUS findings, examined each subject at 38–42 weeks of PMA. The neurological evaluation of the infant was based on the methods of Amiel-Tison: this assessment is divided into six sections and includes 34 items covering cranial assessment, neurosensory function and spontaneous motor activity, passive muscle tone, axial motor activity (active tone), primitive reflexes, palate and tongue, adaptedness to manipulations during assessment, feeding autonomy [21]. Neonatal neurological assessments in preterm infants at term age were classified as “normal” or “abnormal” in the absence or presence of any pathological neurological sign, respectively [21].

### Neurodevelopmental outcome

Each child underwent neurodevelopmental assessment every three months during the first year and every six months in the second year [22]. The Griffiths’ Mental Developmental Scales – Extended Revised (GMDS -ER) [23] were used to obtain child General Quotient (GQ) and the five sub-quotients scores (locomotor, personal-social, hearing and speech, eye-hand coordination, performance) at 24 months of CA. In addition, ophthalmological and audiometric examinations were performed periodically to exclude specific sensory abnormalities [22]. At 24 months of corrected age (CA), infants were classified into three categories according to their neurodevelopmental outcome: “normal” if they had a normal neurological assessment and a GQ ≥ 88; “minor sequelae” if they showed tone and reflex abnormalities or asymmetry without functional deficits, or at least one sign from the triad described by Amiel-Tison and Gosselin [24], GQ between 76 and 87, squint and refractive errors, mild hypoacusia; and “major sequelae” if they presented disabling or non-disabling Cerebral Palsy (CP), a GQ ≤ 75, sensorineural hearing loss requiring active intervention, or severe central or peripheral visual impairment [22].

### Statistical analysis

The continuous variables were reported as mean and standard deviation if normally distributed, as median and interquartile range (IQR: 25th–75th percentile) if not normally distributed. The categorical variables were expressed as counts and percentages.

Comparison between two groups for continuous variables were performed using Student’s t test if normally distributed or the analogous non parametrical test of Mann Whitney if not normally distributed; continuous variables were compared between three groups with the ANOVA test or with the analogous non parametrical Kruskall-Wallis test. Association between categorical variables were studied with the Pearson’s χ^2^ or with the Fisher’s exact test.

Correlation between the scores reported in the GMDS-ER subscales were tested by calculating the Spearman correlation coefficient rho.

Each variable of potential clinical interest was tested in a univariate multinomial (polytomous) regression model in order to assess a potential statistical significance in the association with the outcome sequelae.

The risk factors of clinical (neurological, paediatric, obstetric) interest for which a statistical significance in the association with sequelae at the univariate analysis and for which no collinearity was reported were included as regressors in a multivariate multinomial logistic model.

All tests were two-sided. The significance level was set at α = 0.05. A *p* value < 0.05 was considered statistically significant. Data analysis was performed using the STATA statistical package (version 14 or later; Stata Corporation, College Station, 2009, Texas, USA).

## Results

### Study population

During the study period, two hundred and ninety ELBWi were admitted to the NICU of “Fondazione IRCCS Policlinico San Matteo” hospital. Mean GA at birth was 26.2 (sd 2.4, range 22–33) weeks, and mean birth weight was 750.8 (sd 154.6) g.

Twenty-two infants had a birth weight ≤ 500 g. There were 262 inborn infants (90.3%) while outborn were 28 (9.7%). Two infants were excluded from the analysis due to genetic disorders. Eighty infants (27.6%) died during NICU stay. All infants with birth weight ≤ 500 g died in NICU. GA and BW were significantly lower among infants who died compared with survivors (mean GA at birth 25.1 weeks [sd 2.6 weeks] versus 26.6 weeks [sd 2.2 weeks], *p*-value < 0.001;BW 668 g [sd 164.5 g] versus 782.68 g [sd 138.29 g], *p*-value < 0.001 respectively). None of the surviving infants died after NICU discharge home. Two hundred and eight surviving children met the inclusion criteria and were enrolled. One hundred seventy-six children (84.6%) completed the follow-up evaluation and constituted the study group. No differences in perinatal medical data, cUS findings, or neurological assessment at 40 weeks of PMA were found between the study group and the subjects lost to follow-up.

Table 1 shows the sociodemographic, obstetric and neonatal variables divided by neurodevelopmental outcome. cUS findings and neurological assessment at term age, are reported in Table 1 and Table 2.

**Table 1:** Descriptive statistics for overall sample and distinctly by neurodevelopmental outcome

	***All infants (n* = *176)***	***Normal Outcome (n* = *118)***	***Mild Sequelae (n* = *30)***	***Major Sequelae (n* = *28)***
***Maternal and Obstetric Characteristics***				
Maternal age, mean (sd)	32.02 (5.44)	31.76 (5.12)	32.43 (5.26)	32.65 (7.04)
Maternal Education, yrs N (%)				
≤ 5	3 (3.03)	3 (4.35)	0 (0)	0 (0)
6–8	31 (31.31)	21 (30.43)	6 (33.33)	4 (33.33)
9–13	57 (57.58)	38 (55.07)	12 (66.67)	7 (58.33)
> 13	8 (8.08)	7 (10.14)	0 (0)	1 (8.33)
Preeclampsia N (%)	36 (27.69)	24 (27.59)	8 (34.78)	4 (20)
IUGR N (%)	42 (32.31)	30 (34.48)	10 (43.48)	2 (10)
Cesarean Section, N (%)	134 (76.14)	92 (77.97)	21 (70)	21 (75)
Assisted reproduction, N (%)	25 (14.2)	16 (13.56)	3 (10)	6 (21.43)
Multiple pregnancy, N (%)	35 (19.89)	24 (20.34)	3 (10)	8 (28.57)
Antenatal steroid therapy N (%)				
None	18 (10.23)	9 (7.73)	4 (13.33)	5 (17.86)
Incomplete	134 (76.14)	92 (77.97)	23 (76.67)	19 (67.86)
Complete	22 (12.5)	16 (13.56)	2 (6.67)	4 (14.29)
***Neonatal characteristics***				
Gestational age, mean (sd)	26.52 (2.23)	26.87 (2.27)	26.37 (2.04)	25.18 (1.74)
Male, N (%)	78 (44.32)	46 (38.98)	15 (50)	17 (60.71)
Birthweight, mean (sd)	777.45 (142.89)	800.03 (128.14)	762.93 (124.01)	697.86 (188.79)
SGA, N (%)	45 (25.57)	28 (23.73)	9 (30)	8 (28.57)
Length, mean (sd)	33.18 (2.83)	33.42 (2.3)	32.33 (1.87)	33.12 (4.9)
Head Circumference, mean (sd)	23.76 (2.31)	23.92 (1.96)	23.5 (2.15)	23.38 (3.53)
Stay in NICU, days, mean (sd)	94.49 68.25)	88.27 (71.4)	103.33 (36.09)	111.21 (78.43)
CRIB, mean (sd)	5.19 (3.67)	4.59 (3.33)	5.83 (3.23)	7.04 (4.74)
1-min Apgar score, Median (IQR)	5 (3–7)	5 (4–7)	4 (2–7)	4 (2–6)
5-min Apgar score, Median (IQR)	7 (6–8)	7 (6–8.75)	7 (5.5–9)	7 (5.75–8)
Birth Weight recovery, mean (sd)	13.73 (5)	13.05 (5.23)	14.28 (3.52)	15.86 (4.84)
Resuscitation, N (%)	161 (91.48)	107 (90.68)	26 (86.67)	28 (100)
Bronchopulmonary dysplasia (BPD), N (%)	94 (53.41)	50 (42.37)	21 (70)	23 (82.14)
Postnatal Steroids, N (%)	51 (28.98)	26 (22.03)	14 (46.67)	11 (39.29)
Surgery PDA, N (%)	15 (8.52)	9 (7.63)	2 (6.67)	4 (14.29)
Total Parenteral Nutrition, days, mean (sd)	20.58 (10.35)	19.34 (8.65)	20.27 (10.64)	25.64 (14.25)
ROP grade ≥ 3, N (%)	29 (16.57)	14 (11.97)	6 (20)	9 (32.14)
NEC, N (%)	13 (7.39)	7 (5.93)	2 (6.67)	4 (14.29)
Surgery for NEC, N (%)	6 (3.41)	2 (1.69)	1 (3.33)	3 (10.71)
Early Sepsis, N (%)	1 (.57)	1 (.85)	0 (0)	0 (0)
Late Sepsis, N (%)	58 (32.95)	39 (33.05)	8 (26.67)	11 (39.29)
Normal cUS Findings	21 (11.93)	19 (16.1)	1 (3.33)	1 (3.57)
Mildly Abnormal cUS Findings, N (%)	128 (72.73)	88 (74.58)	25 (83.33)	15 (53.57)
Severely abnormal cUS findings, N (%)	27 (15.34)	11 (9.32)	4 (13.33)	12 (42.86)
Abnormal Neurological examination 40 weeks PMA, N(%)	82 (47.4)	41 (35.34)	20 (68.97)	21 (75)

Maternal and obstetric data are available only for inborn subjects (*n* = 130)

Continuous variables are reported as mean and standard deviation; categorical variables are expressed as count and percentages

**Table 2:** Ultrasound findings and results of neurological assessment at 40 weeks of age

cUS findings classified according to Rademaker, N (%) (*n* = 176)		Neurological Assessment at term equivalent age, N (%) (*n* = 173)	
		Normal	Pathological
Normal, N (%)	21 (11.93%)	15 (71.43%)	6 (28.57%)
Mildly abnormal	128 (72.73%)	68 (53.12%)	57 (44.53%)^a^
*Grade 1 PVL*	*122 (69.32%)*		
*Grade I IVH*	*20 (11.36%)*		
*Grade II IVH*	*5 (2.84%)*		
*Mild Ventricular Dilatation*	*13 (7.39%)*		
Severely abnormal	27 (15.34%)	8 (29.63%)	19 (70.37%)
*Grade II PVL*	*11 (6.25%)*		
*Grade III PVL*	*6 (3.41%)*		
*Grade IV PVL*	*1 (0.57%)*		
*Grade III IVH*	*12 (6.82%)*		
*Grade IV IVH*	*2 (1.14%)*		
*Moderate Ventricular Dilatation*	*12 (6.82%)*		
*Severe Ventricular Dilatation requiring ventricular peritoneal derivation*	*1 (0.57%)*		

^a^neurological assessment data at term age was missing for 3 patients with mildly abnormal cUS findings

### Neurodevelopmental Outcome at 24 months of CA

One hundred eighteen (67.05%) infants showed a neurodevelopmental outcome within the normal range at 24 months of CA. Fifty-eight patients (32.95%) had an “non-optimal” outcome: 30 cases (17.04%) showed minor sequelae and 28 cases (15.91%) major sequelae. In the “major sequelae” group, eight infants (4.54% of the whole sample) had CP, 4 of them with a non-disabling form and could walk unassisted. Severe developmental delay without other associated deficits was observed in 15 infants (8.52% of the sample). Sensory deficits were present in 7 children (2 with visual impairment and 5 with sensorineural hearing loss requiring active intervention). More details are shown in Table 3.

**Table 3:** Neurodevelopmental outcome of the cohort (*n* = 176) at 24 months of CA

Neurodevelopmental Outcome	N (%)
Normal Outcome	118 (67.05%)
Minor sequelae^a^	30 (17.04%)
*76* ≤ *GQ* ≤ *87*	*18 (10.22%)*
*Muscle Tone abnormalities*	*7 (3.97%)*
P*hasic stretch reflex*	*4 (*2.27%)
*Strabismus*	*4 (*2.27%)
*Palpable squamous suture*	*3 (1.7%)*
Major sequelae^a^	28 (15.91%)
*Disabling CP*^b^	*4 (2.27%)*
*Non-disabling CP*^c^	*4 (2.27%)*
*Severe visual Impairment (central or peripheric)*	*2 (*1.14%)
*Hearing impairment needing hearing aid*	5 (2.84%)
*GQ* ≤ *75 without other associated deficits*	15 (8.52%)

*Abbreviations*: *GQ* General quotient, *CP* Cerebral palsy

^a^Each child can present more than one sequela

^b^1 child had GQ < 75, 1 child had GQ < 75 and severe visual impairment, 1 child had GQ < 75 and hearing impairment needing hearing aid

^c^2 children had GQ < 75

**Fig. 2:**
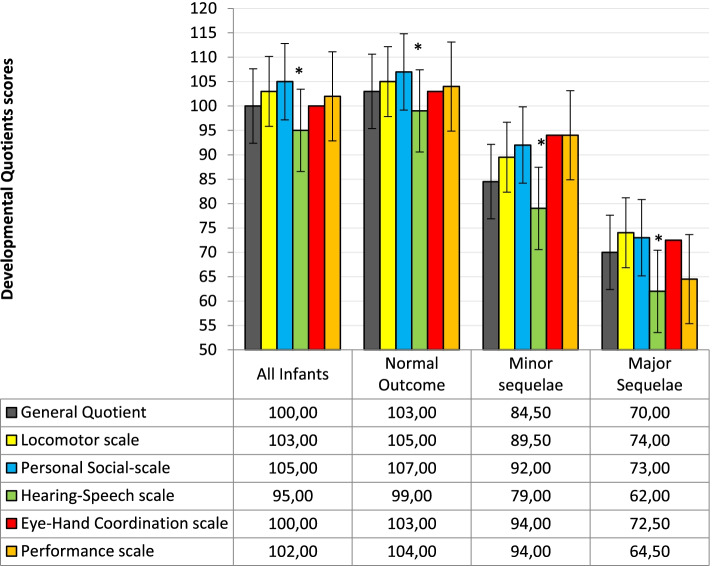
Developmental profile of the entire study sample and divided according to neurodevelopmental outcome at 24 months of CA (median scores)

GMDS-ER scores are available for 174/176 patients. The median GQ was 100 (25th: 88, 75th:106). The median scores obtained on the subscales are shown in Fig. 2. The median score on the Hearing-Speech subscale was significantly lower than the median scores recorded on all the other subscales. Analyzing the pairwise correlation between all the GMDS-ER subscales, the hearing Speech subscale always shows a weaker correlation (rho = 0.48; *p* < 0.001), as reported in Additional Table 1. This weaker correlation together with the statistical significance persisted and was found to be more marked after restricting the analysis to the children with normal GQ (rho = *p* < 0.001), as reported in Additional Table 2.

### Associations between obstetric and neonatal variables and outcome at 24 months of CA

Gestational age was significantly lower in the group with cerebral palsy with 25 (sd = 0.53) week of GA versus 26.59 (sd = 0.17) weeks reported in the group without cerebral palsy; *p* value = 0.048).

Children who reported a GQ score > 75 had significantly higher gestational age (26.71 [sd 0.18] weeks) than children who reported a GQ score ≤ 75 (25.22 [sd 0.37] weeks; *p* value for the comparison = 0.002). Results of comparison of GQ Scale and related subscales between the two groups with different gestational age (< 26 weeks of GA; ≥ 26 weeks of GA) were reported in Additional Table 3, confirming that children < 26 weeks of GA reporting lower mean scores than the children ≥ 26 weeks of GA.

Otherwise, birth weight was significantly associated with GQ score, with children who reported GQ score > 75 showing a mean birth weight equal to 791.01 (sd = 10.4) grams versus a mean birth weight of 687.22 8sd 0 40.82) among children with a GQ score ≤ 75.

Gender was found to be associated with GQ score, with 19.23% (15 males over 78) of males versus 8.16% (8 females over 98) of females reporting GQ score ≤ 75 (*p* value for the comparison = 0.030).

Table 4 reports results of both univariate and multivariate analysis of risk factors associated with neurodevelopmental sequelae.

**Table 4:** Univariate and multivariate analysis of risk factors for adverse neurodevelopmental outcome

Variable	Univariate Analysis						Multivariate Analysis					
	Minor Sequelae			Major Sequelae			Minor Sequelae			Major Sequelae		
	RRR	CI 95%	*p*	RRR	CI 95%	*p*	RRR	CI 95%	*p*	RRR	CI 95%	*p*
Female Sex	0.64	0.29–1.43	0.276	0.41	0.18–0.96	0.040*						
Birthweight (g)	1	1–1	0.187	0.99	0.99–1.00	0.001*						
Gestational age (weekd)	0.90	0.74–1.08	0.265	0.66	0.52–0.83	0.001*						
Bronchopulmonary dysplasia (BPD)	3.17	1.34–7.51	0.009*	6.26	2.23–17.59	0.001*	2.95	1.19–7.33	0.020*	4.36	1.48–12.86	0.008*
Postnatal steroid	3.10	1.34–7.16	0.008*	2.29	0.95–5.49	0.063						
Red blood cells transfusion	1.43	0.53–3.81	0.480	4.63	1.04–20.67	0.045*						
CRIB	1.10	0.99–1.23	0.084	1.19	1–07-1.34	0.002*						
Birthweight recovery (days)	1.06	0.97–1.15	0.251	1.12	1.03–1.21	0.010*	1.03	0.95–1.13	0.426	1.06	0.97–1.17	0.186
Total Parenteral Nutrition (days)	1.01	0.97–1.06	0.654	1.06	1.01–1.10	0.011*						
cUS findings:												
Mildly abnormal	5.40	0.69- 42–32	0.109	3.24	0.40–26.03	0.269						
Severely abnormal	6.91	0.68–69.86	0.102	20.73	2.36–181.71	0.006*	4.03	0.38–43.14	0.249	10.22	1.08–96.78	0.043*
Abnormal neurological examination at 40 wks PMA	4.07	1.70–9.74	0.002*	5.49	2.15–14.00	< 0.001*						

*p* values < 0.05 indicating statistical significance of the association between the variable under study and the neurological sequelae are highlighted with an asterisk

Severely abnormal cUS findings and bronchopulmonary dysplasia (BPD) were the most important predictors of major sequelae (respectively RRR = 20.73 – *p* value = 0.006 for severely abnormal cUS findings; RRR = 6.26 – *p* value = 0.001 for BPD).

Abnormal neurological examination at 40 weeks PMA (scoring based on the methods of Amiel-Tison and Gosselin [21, 24], including pathological signs of variable degree, in particular no fix and track, abnormal spontaneous activity, excessive neck extensor muscles response) degree and red blood cell transfusion were found to be statistically significant risk factors for major sequelae, with a weaker association than the ones aforementioned (respectively RRR = 5.49 – *p* value < 0.001; 4.63 – *p* value = 0.045). Pathological neurological examination at term age has been identified as a moderate risk factor for minor sequel too.

Further weak association, even if with statistical significance of the association with major sequelae, was reported for CRIB, time needed for birthweight recovery and total parenteral nutrition (respectively RRR = 1.19 – *p* value = 0.002; RRR = 1.12 – *p* value = 0.010; RRR = 1.06; *p* value = 0.011). In contrast, female gender resulted as a moderate protective factor for major sequelae (RRR = 0.41- *p* value = 0.040), as well as a greater gestational age (RRR = 0.66; *p*-value = 0.001) Post-natal steroid administration is a moderate risk factor only for minor sequelae (RRR 3.10 *p* = 0.008).

The multivariate model was performed by including the variables of clinical interest for which no collinearity was reported.

Multivariate multinomial logistic regression analysis confirmed severely abnormal cUS findings and BPD as independent risk factors significantly associated to major sequelae (respectively RRR = 10.22—*p* value = 0.043 for severely abnormal cUS findings; RRR = 4.36 – *p* value = 0.008 for BPD) and only BPD as predictor for minor sequelae (RRR = 2.95; *p* value = 0.020).

## Discussion

This study investigated neurodevelopmental outcomes at two-years corrected age of an Italian single tertiary care center cohort of ELBW infants born between 2005 and 2015.

Adverse neurodevelopmental outcomes involved about 33% of our sample, half of which were minor sequelae. The most frequent major sequela was cognitive impairment.

Two-third of survivors had no neurodevelopmental impairment at 24 months of CA.

Results from previous international ELBW cohorts showed higher rates of severe disability, when definition criteria for severe disability were comparable to those applied in our cohort [6, 7, 24].

The most recent EPIPAGE-2 study showed results more similar to ours even though infants were classified according to gestational age rather than birthweight (survival rates without neurodevelopmental impairment at 2 years of CA of 48.5%, 90.0%, and 97.5% for children born at 22–26, 27–31, and 32–34 weeks’ gestation respectively) [25]. Moreover, the survival rate without developmental disabilities of our cohort of ELBW was similar to the most recent studies, with a prevalence of mild over major sequelae compared to previous ones, as shown in a recent Chinese study where 64% of infants with GA between 22 and 28 weeks survived without neurodevelopmental disabilities at 18–24 months of CA [26].

Several reasons could explain differences in neurodevelopmental impairment rates and severity. First, inclusion criteria are based on gestational age or birth weight; even though most ELBW infants are preterm, gestational age may vary depending on the number of small for gestational age infants enrolled. Indeed, fetal growth restriction is a well-known risk factor for neurodevelopmental impairment [27]. Therefore, one possible explanation for the higher percentage of infants without severe disabilities observed in our population, compared to previous studies, may be related to the lower rate of intrauterine growth-restricted newborns from our cohort. Moreover, neurodevelopmental disability definition criteria may vary, depending on the assessment tests.

Another explanation of results discrepancies among studies may rely on the recruitment period. Indeed, our cohort includes infants born between 2005 and 2015, while others were based on cohorts enrolled between 1998- and 2007 [6, 7, 28],, whereas EPIPAGE-2 [9] and the Chinese study group [26] were more recent (2011 and 2016 respectively).

Assuming that in the last decade improvement in antenatal, perinatal and postnatal care has led to neurodevelopmental outcome improvement, we may argue that more recent case studies might reflect clinical progression of the management.

Differently, the CP prevalence reduction demonstrated for VLBWI [29, 30] is not confirmed for ELBW infants, likely due to the higher survival rate of the most preterm newborns (i.e. 23–24 gestational ages), actually ranging from 7 to 20% [9, 31–33].

To our knowledge, a comparable CP rate (around 5%) was described by Wilson-Costello et al. only. [34]

Another point to underline is that our sample's most frequent major sequela was a developmental delay (8.52%). These data align with those recently reported by an Italian study in which 9.1% of ELBW infants showed a cognitive score below 2SD at 24 months of corrected age [35]. Other recent international studies reported higher rates of cognitive impairment, up to 30%, especially for infants with a birth weight ≤ 750 g, which significantly reduce in infants with birth weight ≥ 750 g [36].

Regarding GQ values and developmental subscale scores, we found a peculiar profile maintained regardless of normal GQ and characterized by a significant selective deficit in the language domain [37]. A left-shift of the “normal” distribution curves for language and cognition has been described for children born very preterm [38] and abnormal speech ratings remained significantly higher among ELBW infants after adjustment for cognition [39].

Several studies suggested that language difficulties in preterm children are not a mere expression of co-occurring cognitive disabilities; instead, they seem to emerge as complications of prematurity that may selectively interfere with language acquisition [40].

Recent studies also highlighted that prematurity is associated with an increased risk for autism spectrum disorders [41]. At older ages, preterm infants’ emotional and behavioral phenotype was described [42]. The methodology and tools used in our study do not allow us to precisely quantify how many and which children are at risk of developing an autism spectrum disorder or social-emotional dysregulation. However, we are aware that the impaired GQ scores on the developmental test reflect both the fragility of the cognitive process and the presence of emotional dysregulation with social communication and interaction problems and sometimes repetitive behaviors.

As previously reported [18], severely abnormal cUS findings, especially periventricular leukomalacia, are strong predictors of major sequelae.

Cerebral US is the standard bedside examination in NICU. Despite its limits in identifying specific types of brain damage, such as diffuse white matter abnormalities and micro-cerebellar hemorrhages, our results confirmed its importance in clinical practice in defining the neurological prognosis in the short term and referring infants to specialized early intervention programs.

Our sample identified bronchopulmonary dysplasia as the other independent risk factor for both minor and major neurodevelopmental sequelae.

Children who developed BPD are at increased risk of brain injury‍ and abnormal brain development [43, 44]. It is still unknown the pathophysiologic mechanism linked to neurodevelopmental impairment in BPD patients and to what extent other factors associated with immaturity and or BPD, such as oxygen dependency, blood gas derangements‍, systemic inflammation or immature autoregulation system determined neurodevelopmental impairment [43, 45].

Moreover, we found that the time needed to recover birth weight and red blood cells transfusion were other risk factors associated with impaired neurodevelopment, although only at univariate analysis.

One possible explanation for the relationship between delayed time to birth weight recovery and impaired neurodevelopment could rely on a possible inadequate amount of specific macro and micronutrients during the critical phases of neurogenesis, neuronal differentiation, myelination and synaptogenesis [46]. However, to our knowledge, despite the great interest in nutrition effects on brain development, few studies have investigated this specific factor [28, 47].

It is already reported that RBC transfusion negatively impacts the long-term neurodevelopment of preterm newborns [48]. In the clinical setting of an underlying inflammatory condition, such as prematurity, blood transfusions may trigger immune cell activation and the pro-inflammatory response of the endothelial and the immune system, promoting cytokines release. Moreover, transfused preterm newborns might be exposed to a potential source of neurotoxic metals.

### Strength and limitations

Our study is one of the few investigating the neurodevelopmental outcome at two years of corrected age of ELBWi born in the 2000s in Italy [22, 49]. Overall, 80% of enrolled participants completed the follow-up, a cut-off used to define high-quality randomized trials [50].

The single-center design of the study meant that highly homogeneous standards of care were applied to all participants throughout the study period and ensured methodological consistency of data collection during the follow-up.

In this retrospective study obstetrical data are complete and available only for a subgroup of infants. Among incomplete maternal data, there were maternal emotional well-being and stress perception, stressful life events, depressive symptoms and pregnancy-related anxiety, known to be associated with neurodevelopmental outcomes in extremely preterm infants [51].

Additionally, the predictive value of neurological examination improves with the analysis of GMs’ trajectories; however, video-recordings with the strict methodology needed for research are difficult to obtain for every patient in routine clinical settings. Nevertheless, the quality of spontaneous motility has been evaluated during the neurological examination performed at 40 weeks PMA. Unfortunately, we do not have neonatal brain MRI data available for all patients: it would be interesting to evaluate them, in particular in the proportion of children with cognitive delay at 24 months of corrected age.

## Conclusion

Our study showed an improvement of ELBW infants' survival rate without neurodevelopmental impairment compared to previous studies and a tendency to change the clinical type of major sequelae, with a prevalence of cognitive impairment on cerebral palsy. Even though the follow-up period until 24 months corrected age can identify major sequelae, long-term follow-up is mandatory for ELBW infants because neuropsychological and behavioral deficits related to preterm birth are ultimately expressed only at older ages. Finally, standardized follow-up protocols to detect and quantify infants with emotional dysregulation and risk of developing autism spectrum disorder are required.

## Supplementary Information

**Additional file 1: Table S1.** Correlation between GMDS-ER subscales scores reported for overall sample (*n*=176). rho Spearman correlation coefficient is reported in the first line of each cell; in the second line of each cell *p* value for the statistical significance of the correlation is shown.

**Additional file 2: Table S2.** Correlation between GMDS-ER subscales scores reported for children with normal GQ score (*n*=153). rho Spearman correlation coefficient is reported in the first line of each cell; in the second line of each cell *p* value for the statistical significance of the correlation is shown.

**Additional file 3: Table S3.** Comparison of GMDS-ER GQ and related subscales between the two groups with different gestational age (<26 weeks of GA; ≥ 26 weeks of GA). Data are reported as mean (standard deviation).

## Data Availability

The datasets used and/or analysed during the current study are available from the corresponding author on reasonable request.

## Abbreviations

ELBW: Extremely low birth weight
CA: Corrected age
NICU: Neonatal intensive care unit
VON: Vermont Oxford Network
cUS: Cranial ultrasound
IVH: Intraventricular hemorrhage
PVL: Periventricular leukomalacia
VD: Ventricular dilatation
GMDS-ER: Griffiths’ Mental Developmental Scales – Extended Revised
GA: Gestational age
CP: Cerebral palsy
BW: Birth Weight
IUGR: Intrauterine Growth restriction

## References

[1] Blencowe H, Cousens S, Chou D (2013). Born Too Soon : The global epidemiology of 15 million preterm births. Reprod Health.

[2] Hug L, Alexander M, You D, Alkema L (2019). National, regional, and global levels and trends in neonatal mortality between 1990 and 2017, with scenario-based projections to 2030: a systematic analysis. Lancet Glob Heal.

[3] Twilhaar ES, Wade RM, De Kieviet JF, Van Goudoever JB, Van Elburg RM, Oosterlaan J (2018). Cognitive outcomes of children born extremely or very preterm since the 1990s and associated risk factors: a meta-analysis and meta-regression. JAMA Pediatr.

[4] Van Lieshout RJ, Ferro MA, Schmidt LA (2018). Trajectories of psychopathology in extremely low birth weight survivors from early adolescence to adulthood: a 20-year longitudinal study. J Child Psychol Psychiatry Allied Discip.

[5] Moore T, Hennessy EM, Myles J, et al. Neurological and developmental outcome in extremely preterm children born in england in 1995 and 2006: The epicure studies. BMJ. 2012;345. doi:10.1097/01.ogx.0000429293.80760.f2 10.1136/bmj.e7961 PMC3514471 23212880

[6] Mercier CE, Dunn MS, Ferrelli KR, Howard DB, Soll RF (2010). Neurodevelopmental outcome of extremely low birth weight infants from the vermont oxford network: 1998–2003 and the Vermont Oxford Network ELBW infant follow-up study group. Neonatology.

[7] NICHD Neonatal Research Network (2013). Characteristics of extremely low birth weight infant survivors with unimpaired outcomes at 30 months of age. J Perinatol.

[8] Serenius F, Källén K, Blennow M, EG (2013). Neurodevelopmental outcome in extremely preterm infants at 2.5 years after active perinatal care in Sweden. JAMA.

[9] V-M Pierrat L Arnaud C Kaminski M Resche-Rigon M Lebeaux C Bodeau-Livinec F MorgaN A Goffinet F Marret S Ancel PE-2 writing group 2017 Neurodevelopmental outcome at 2 years for preterm children born at 22 to 34 weeks’ gestation in France in, 2011 EPIPAGE-2 cohort study BMJ 358 j3448 10.1136/bmj.j3448 PMC5558213 28814566

[10] Brumbaugh JE, Hansen NI, Bell EF (2019). Outcomes of extremely preterm infants with birth weight less than 400 g. JAMA Pediatr.

[11] Inoue H, Ochiai M, Sakai Y (2018). Neurodevelopmental outcomes in infants with birth weight ≤500 g at 3 years of age. Pediatrics.

[12] Lugli L, Bedetti L, Guidotti I (2021). Neuroprem 2: an italian study of neurodevelopmental outcomes of very low birth weight infants. Front Pediatr.

[13] Spittle, A Orton, J Anderson, PJ Boyd, R Doyle L. Early developmental intervention programmes provided post hospital discharge to prevent motor and cognitive impairment in preterm infants. Cochrane DatabaseSystRev. 2015;11. 10.1002/14651858.CD005495.pub4 PMC8612699 26597166

[14] Horbar J (1999). The Vermont Oxford Network: evidence-based quality improvement for neonatology. Pediatrics.

[15] Gagliardi L (2004). Assessing mortality risk in very low birthweight infants: a comparison of CRIB, CRIB-II, and SNAPPE-II. Arch Dis Child Fetal Neonatal Ed.

[16] Leijser LM, de Vries LS, Cowan F (2006). Using cerebral ultrasound effectively in the newborn infant. Early Hum Dev.

[17] Papile LA, Burstein J, Burstein R (1978). Incidence and evolution of subependymal and intraventricular hemorrhage: a study of infants with birth weights less than 1,500 g. J Pediatr..

[18] De Vries LS, Van Haastert IL, Rademaker KJ, Koopman C, Groenendaal F (2004). Ultrasound abnormalities preceding cerebral palsy in high-risk preterm infants. J Pediatr..

[19] Ment LR, Vohr B, Allan W (1999). The etiology and outcome of cerebral ventriculomegaly at term in very low birth weight preterm infants. Pediatr Neonatol..

[20] Rademaker KJ, Uiterwaal CSPM, Beek FJA (2005). Neonatal cranial ultrasound versus MRI and neurodevelopmental outcome at school age in children born preterm. Arch Dis Child Fetal Neonatal Ed.

[21] Amiel-Tison C (2002). Update of the Amiel-Tison neurological assessment for the term neonate or at 40 weeks corrected age. Pediatr Neurol.

[22] Olivieri I, Bova SM, Urgesi C (2012). Outcome of extremely low birth weight infants: What’s new in the third millennium? Neuropsychological profiles at four years. Early Hum Dev.

[23] Griffiths, R Huntley M. The Griffiths Mental Development Scales-Revised Manual: From Birth to 2 Years. (ARICD, ed.). High Wycombe; 1996.

[24] Gosselin J, Gahagan S, Amiel-Tison C (2005). The Amiel-Tison Neurological Assessment at Term: Conceptual and methodological continuity in the course of follow-up. Ment Retard Dev Disabil Res Rev.

[25] Pierrat V, Marchand-Martin L, Arnaud C, et al. Neurodevelopmental outcome at 2 years for preterm children born at 22 to 34 weeks’ gestation in France in 2011: EPIPAGE-2 cohort study. BMJ. 2017;358. 10.1136/bmj.j3448 10.1136/bmj.j3448 PMC5558213 28814566

[26] Li Y, GCRG for EPI (2019). Neurodevelopmental outcomes of extremely preterm infants in southern China: A multicenter study. Early Hum Dev.

[27] Levine TA, Grunau RE, McAuliffe FM, Pinnamaneni R, Foran A, Alderdice F (2015). Early childhood neurodevelopment after intrauterine growth restriction: a systematic review. Pediatrics..

[28] Maruyama H, Yonemoto N, Kono Y (2015). Weight growth velocity and neurodevelopmental outcomes in extremely low birth weight infants. Plos One.

[29] Platt MJ, Cans C, Johnson A (2007). Trends in cerebral palsy among infants of very low birthweight (<1500 g) or born prematurely (<32 weeks) in 16 European centres: a database study. Lancet.

[30] Sellier E, Platt MJ, Andersen GL (2016). Decreasing prevalence in cerebral palsy: a multi-site European population-based study, 1980 to 2003. Dev Med Child Neurol.

[31] Platt MJ, Cans C, Johnson A, Surman G, Topp M, Torrioli MG, Krageloh-Mann I (2007). Trends in cerebral palsy among infants of very low birthweight (<1500 g) or born prematurely (<32 weeks) in 16 European centres: a database study. Lancet.

[32] Roberts G, Anderson PJ, De Luca C, Doyle LW (2010). Changes in neurodevelopmental outcome at age eight in geographic cohorts of children born at 22–27 weeks’ gestational age during the 1990s. Arch Dis Child Fetal Neonatal Ed.

[33] Hafström M, Källén K, Serenius F, et al. Cerebral palsy in extremely preterm infants. Pediatrics. 2018;141(1). doi:10.1542/peds.2017-1433 10.1542/peds.2017-1433 29222398

[34] Wilson-Costello D, Friedman H, Minich N (2007). Improved Neurodevelopmental outcomes for extremely low birth weight infants in 2000–2002. Pediatrics.

[35] Gardon L, Picciolini O, Squarza C (2018). Neurodevelopmental outcome and adaptive behaviour in extremely low birth weight infants at 2 years of corrected age. Early Hum Dev.

[36] B Courchia MD Berkovits CR Bauer Cognitive impairment among extremely low birthweight preterm infants from, 1980 to present day J Perinatol 2019 1098 1104 10.1038/s41372-019-0414-x 10.1038/s41372-019-0414-x 31235783

[37] Orcesi S, Olivieri I, Longo S (2012). Neurodevelopmental outcome of preterm very low birth weight infants born from 2005 to 2007. Eur J Paediatr Neurol.

[38] Roberts G, Anderson PJ, De Luca C, Doyle L (2010). Changes in neurodevelopmental outcome at age eight in geographic cohorts of children born at 22-27 weeks’ gestational age during the 1990s. Arch Dis Child Fetal Neonatal.

[39] Vohr BR (2016). Language and hearing outcomes of preterm infants. Semin Perinatol.

[40] De Stefano P, Marchignoli M, Pisani F, Cossu G. Uneven Linguistic Outcome in Extremely Preterm Children. J Psycholinguist Res. 2019;(0123456789). doi:10.1007/s10936-019-09662-x 10.1007/s10936-019-09662-x 31407217

[41] Cogley, C, O’Reilly, H Bramham J et al. A Systematic Review of the Risk Factors for Autism Spectrum Disorder in Children Born Preterm. Child Psychiatry Hum Dev. 2020. doi:10.1007/s10578-020-01071-9. 10.1007/s10578-020-01071-9 32980936

[42] Burnett AC, Youssef G, Anderson P, VICSG (2019). Exploring the “preterm behavioral phenotype” in children born extremely preterm. J Dev Behav Pediatr.

[43] Sriram S, Schreiber MD, Msall ME (2018). Cognitive development and quality of life associated with BPD in 10-year-olds born preterm. Pediatrics.

[44] Neubauer V, Junker D, Griesmaier E, Schocke M, Kiechl-Kohlendorfer U (2015). Bronchopulmonary dysplasia is associated with delayed structural brain maturation in preterm infants. Neonatology.

[45] Albertine K (2012). Brain injury in chronically ventilated preterm neonates: collateral damage related to ventilation strategy. Clin Perinatol.

[46] Volpe JJ (2019). Dysmaturation of premature brain : importance, cellular mechanisms, and potential interventions. Pediatr Neurol.

[47] Verma R, Shibly S, Fang H, Pollack S (2015). Do early postnatal body weight changes contribute to neonatal morbidities in the extremely low birth weight infants. J Neonatal Perinatal Med.

[48] Fontana, C Raffaeli, G Pesenti, N Boggini, T Cortesi, V Manzoni, F Picciolini, O Fumagalli, M Mosca, F Ghirardello S. Red blood cell transfusions in preterm newborns and neurodevelopmental outcomes at 2 and 5 years of age.tle. Blood Transfus. 2020;1. doi:10.2450/2020.0207-20. 10.2450/2020.0207-20 PMC8796841 33263525

[49] Uccella S, De Carli A, Sirgiovanni I (2015). Survival rate and neurodevelopmental outcome of extremely premature babies: An 8-year experience of an Italian single neonatal tertiary care center. Pediatr Medica e Chir.

[50] Fewtrell MS, Kennedy K, Singhal A (2008). How much loss to follow-up is acceptable in long-term randomised trials and prospective studies?. Arch Dis Child..

[51] Ylijoki MK, Ekholm E, Ekblad M, Lehtonen L (2019). Prenatal risk factors for adverse developmental outcome in preterm infants-systematic review. Front Psychol.

