# Supplementary material for: Neurodevelopmental outcome of Italian preterm ELBW infants: an eleven years single center cohort
Source: Ital J Pediatr. 2022 Jul 19;48:117. doi: 10.1186/s13052-022-01303-9 (PMC9297614; doi:10.1186/s13052-022-01303-9)
Supplement: Supplementary file 1 — Additional file 1: Table S1. Correlation between GMDS-ER subscales scores reported for overall sample (n=176). rho Spearman correlation coefficient is reported in the first line of each cell; in the second line of each cell p value for the statistical significance of the correlation is shown. [file 13052_2022_1303_MOESM1_ESM.docx]

**Additional Table1** Correlation between GMDS-ER subscales scores reported for overall sample (n=176)

| **GMDS-ER subscales** |  | *Locomotor* | *Personal social* | *Hearing Speech* | *Eye-hand coordination* |
| --- | --- | --- | --- | --- | --- |
| *Personal social* | rho | 0.642  <0.0001 |  |  |  |
|  | p value |  |  |  |  |
| *Hearing speech* | rho | 0.483  <0.0001 | 0.662  <0.0001 |  |  |
|  | p value |  |  |  |  |
| *Eye-hand coordination* | rho | 0.574  <0.0001 | 0.613  <0.0001 | 0.566  <0.0001 |  |
|  | p value |  |  |  |  |
| *Performance* | rho | 0.562  <0.0001 | 0.589  <0.0001 | 0.596  <0.0001 | 0.670  <0.0001 |
|  | p value |  |  |  |  |

*rho Spearman correlation coefficient is reported in the first line of each cell; in the second line of each cell p value for the statistical significance of the correlation is shown.*
