# Supplementary material for: Neurodevelopmental outcome of Italian preterm ELBW infants: an eleven years single center cohort
Source: Ital J Pediatr. 2022 Jul 19;48:117. doi: 10.1186/s13052-022-01303-9 (PMC9297614; doi:10.1186/s13052-022-01303-9)
Supplement: Supplementary file 2 — Additional file 2: Table S2. Correlation between GMDS-ER subscales scores reported for children with normal GQ score (n=153). rho Spearman correlation coefficient is reported in the first line of each cell; in the second line of each cell p value for the statistical significance of the correlation is shown. [file 13052_2022_1303_MOESM2_ESM.docx]

**Additional Table 2** Correlation between GMDS-ER subscales scores reported for children with normal GQ score (n=153)

| **GMDS-ER subscales** |  | *Locomotor* | *Personal social* | *Hearing Speech* | *Eye-hand coordination* |
| --- | --- | --- | --- | --- | --- |
| *Personal social* | rho | 0.527  <0.0001 |  |  |  |
|  | p value |  |  |  |  |
| *Hearing speech* | rho | 0.346  <0.0001 | 0.547  <0.0001 |  |  |
|  | p value |  |  |  |  |
| *Eye-hand coordination* | rho | 0.429  <0.0001 | 0.458  <0.0001 | 0.411  <0.0001 |  |
|  | p value |  |  |  |  |
| *Performance* | rho | 0.422  <0.0001 | 0.427  <0.0001 | 0.427  <0.0001 | 0.542  <0.0001 |
|  | p value |  |  |  |  |

*rho Spearman correlation coefficient is reported in the first line of each cell; in the second line of each cell p value for the statistical significance of the correlation is shown.*
