# Supplementary material for: Neurodevelopmental outcome of Italian preterm ELBW infants: an eleven years single center cohort
Source: Ital J Pediatr. 2022 Jul 19;48:117. doi: 10.1186/s13052-022-01303-9 (PMC9297614; doi:10.1186/s13052-022-01303-9)
Supplement: Supplementary file 3 — Additional file 3: Table S3. Comparison of GMDS-ER GQ and related subscales between the two groups with different gestational age (<26 weeks of GA; ≥ 26 weeks of GA). Data are reported as mean (standard deviation). [file 13052_2022_1303_MOESM3_ESM.docx]

**Additional Table 3.**

Comparison of GMDS-ER GQ and related subscales between the two groups with different gestational age (<26 weeks of GA; ≥ 26 weeks of GA)

|  | *GA < 26 weeks* | *GA ≥ 26 weeks* | *p value* |
| --- | --- | --- | --- |
| *General Quotient Score* | 90.42 (18.34) | 99.07 (14.9) | 0.002 |
| *Locomotor* | 92.90 (21.88) | 100.90 (17.67) | 0.009 |
| *Personal social* | 93.56 (19.76) | 102.45 (17.64) | 0.0006 |
| *Hearing speech* | 83.84 (21.79) | 92.87 (17.71) | 0.009 |
| *Eye-hand coordination* | 91.68 (17.71) | 100.53 (15.42) | 0.0003 |
| *Performance* | 90.99 (21.87) | 100.53 (18.94) | 0.01 |

*Data are reported as mean (standard deviation)*
